# Progress in Surgery

**Published:** 1895-03-23

**Authors:** 


### PROGRESS IN SURGERY.

### ABDOMINAL SURGERY.

**The Incision in Abdominal Surgery** is so often followed by ventral hernia when closed *en masse* by the ordinary suture, that Carstens[1] considers the following method best, and states that hernia never follows its adoption: 1. In closing the abdominal incision use animal ligature, kangaroo tendon or catgut. First carefully bring together the peritoneum in a running stitch, then the transversalis fascia, and the rectus, if the incision is through this muscle. Then carefully bring together, edge to edge, the tendinous insertion of the oblique muscles. The loose cellular tissue and fat can be brought together in one or two tiers, according to thickness. Bring the skin carefully together with Marcy's cobbler's stitch, thus burying all the sutures. The incision is then sealed with collodion. 2. In cases of extensive umbilical, ventral or other hernia, it is best to bring the peritoneum together with an over and over stitch of kangaroo tendon or catgut; to make a flap splitting operation of the ring, which is brought together with silkworm gut or silver wire, which is buried, and then the fat and skin are united with the buried animal suture.

The apposition of peritoneum to peritoneum is a surgical error, according to Greig Smith's experience,[2] and he believes that a sero-fibrous union is much firmer than a fibro-fibrous or sero-serous union.

**The Cause of Thirst following Abdominal Sections** is believed by Boise[3] to be due to the direct and reflex irritation of the abdominal sympathetic nerves. Such irritation causes contraction, in some degree, of the arterioles of the abdominal viscera, with consequent lessened tension in their capillaries and compensatory withdrawal of water from their tissues. It is this withdrawal of fluid which gives rise to the sensation of thirst.

**Painful Peritoneal Adhesions** may, says Nicaise,[4] give trouble by disturbing the functions of implicated organs, and by exciting pain. Diagnosis is often difficult, and in some cases their existence can only be assumed by a process of exclusion. In many cases, however, a diagnosis can be made by inquiry concerning such details as the previous occurrence of abdominal inflammation, the seat of the pain and its relation to that of previous inflammatory attacks, the time when the pain comes on with regard to food, menstruation, &c. As such adhesions tend to become longer and thinner, we should not be in a hurry to operate unless they cause very severe and frequently-renewed pain. Laparotomy is the best means of dealing with them, and, as a rule, the cure is complete and permanent. Professor Riedel,[5] as a result of twenty-one original observations, takes much the same view; but, although admitting the success of the immediate results, is inclined to think that the adhesions are reproduced in the course of time.

**Peritonitis.**—Howard Marsh[6] reports a successful case of acute general peritonitis following enteritis, in which he performed laparotomy, then opened the bowel and removed a large amount of flatus and fæcal material, and after stitching up the bowel irrigated the abdominal cavity. This line of treatment was adopted because it was believed that the peritonitis was caused by infection from the intestine. Picqué[7] has had a rare case of encysted purulent peritonitis with pneumococci simulating appendicitis. Microscopical examination of the pus gave evidence of the existence of diplococci and bacilli in the form of short chains. Some of these micro-organisms were surrounded by a distinct capsule, and Dr. Brault considered them to be pneumococci. It appears that no case of this kind has ever been reported in an adult. Lockwood,[8] in speaking of diffuse septic peritonitis, pointed out the importance of intestinal paralysis, and distension, in producing the fatal result. In the author's successful cases a systematic attempt was made to meet exhaustion and collapse by stimulating the patient temporarily before the operation with strychnine and brandy, by the application of warmth during the operation, by rapid and methodical operating, and by warmth, stimulants and nutrient enemata afterwards. The treatment of the distended intestines is important. To empty them, the gas was drawn from each coil by puncturing it with a fine trocar and cannula; the fæces were let out by an incision which was afterwards closed. The abdomen was then irrigated and closed after the insertion of a drain. Two cases were described in which the treatment was successful. In the first there was a perforation of the ileum, and the peritonitis was of not less than forty-eight hours' standing. In the second case the peritonitis had existed about fifty-two hours, and was thought to have been due to a septic inflammation of the left Fallopian tube. The abdomen was full of free gas, with the usual lymph and purulent fluid. Herhold[9] also relates two cases in which recovery ensued after incision and drainage. Of twenty-five such cases which have occurred in the Berlin Charité, five have recovered.

**Tuberculous Peritonitis.**—Frees[10] mentions the fact that operation statistics show a great preponderance of women, whereas pathological registers disclose a predominance of men. The effusive form is usually looked upon as more favourable than the adhesive or obliterating. The author gives details of eighteen cases of tuberculous peritonitis with effusion treated by abdominal section, of which six were completely cured. Nodules were found in all the cases on the visceral and parietal peritoneum. As regards the operative procedure, as small an incision as possible is made in the linea alba, a digital exploration carried out, and the fluid evacuated. Some were drained for the first two days, but no difference was noted from those not so drained. In no case was death due to the operation. Stchégoleff[11] thinks that the actual curative influence of cœliotomy is probably due to traumatism of the peritoneum, thermal action, and the effect of air and light, the combined effect of these factors being a peritoneal irritation, followed by a more or less intense inflammatory reaction, which arrests the morbid process. The tissues around the tubercles become infiltrated with embryonic cells, which constitute a barrier to the extension of the focus and attack the bacilli; undergoing organization, they are transformed into fibrous tissue, the specific germs being eventually destroyed and absorbed. The author thinks that the effect of abdominal section is equally good in cases in which there is no fluid.

Marfan[12] considers that surgical treatment embraces puncture, with or without injection of medicated solutions, and laparotomy. Simple puncture is indicated in cases of an abundant ascites which show no tendency to recede under medical treatment. In encysted collections puncture is useful as a method of exploration, and sometimes, though exceptionally, curative. The injection, after evacuation, of boric acid solution (Debove), *naphtol camphré* (Rendu), blood-serum of dogs (Kirmisson, Pinard), and of sterilized air (Von Mosetig-Moorhof) have given little encouragement. Laparotomy is useful in fibro-caseous peritonitis with ascites, encysted peritonitis, and intestinal occlusion occurring in the course of a tubercular peritonitis. Advanced tuberculous lesions elsewhere, and chronic albuminuria, whether tubercular or not, are contra-indications to operation. Guignabert[13] says he has employed Rendu's treatment (injection of camphorated naphthol) with success.

[1] Amer. Journ. of Obstet., Oct., 1894. [2] Brit. Med. Journ., Jan. 5, 1895, p. 1. [3] Amer. Journ. of Obstet., Oct. 1894, p. 484. [4] Brit. Med. Journ., Oct. 20, 1894, and Rev. de Chir., Aug., 1894. [5] Med. Week, Dec. 14, 1894. [6] Lancet, Oct. 27, 1894, p. 973. [7] Med. Week, Oct. 12, 1894, p. 503. [8] Brit. Med. Journ., and Lancet, Oct. 27, 1894. [9] Brit. Med. Journ., Nov. 3, 1894, and Deut. Med. Woch., Sept. 27, 1894. [10] Brit. Med. Journ., Dec. 8, 1894, and Deut. Med. Woch., 1894, Nos. 45 and 46. [11] Amer. Journ. Med. Sci., Nov. 1894, and Arch. de Med. experiment. et d'Anat. path., Sept. 1, 1894. [12] Amer. Journ. Med. Sci., Nov., 1894, and La Presse, Med,, Aug. 18, 1894. [13] Practitioner, Aug., 1894, p. 126, and Journ. de Med. de Paris, 1894, p. 155.

